# Crystal Structure of the *N*-Acetyltransferase Domain of Human *N*-Acetyl-L-Glutamate Synthase in Complex with *N*-Acetyl-L-Glutamate Provides Insights into Its Catalytic and Regulatory Mechanisms

**DOI:** 10.1371/journal.pone.0070369

**Published:** 2013-07-24

**Authors:** Gengxiang Zhao, Zhongmin Jin, Norma M. Allewell, Mendel Tuchman, Dashuang Shi

**Affiliations:** 1 Center for Genetic Medicine Research and Department of Integrative Systems Biology, Children’s National Medical Center, The George Washington University, Washington, D. C., United States of America; 2 Southeast Regional Collaborative Access Team, Advanced Photon Source, Argonne National Laboratory, Argonne, Illinois, United States of America; 3 Department of Cell Biology and Molecular Genetics and Department of Chemistry and Biochemistry, College of Computer, Mathematical, and Natural Sciences, University of Maryland, College Park, Maryland, United States of America; University of Melbourne, Australia

## Abstract

*N*-acetylglutamate synthase (NAGS) catalyzes the conversion of AcCoA and L-glutamate to CoA and *N*-acetyl-L-glutamate (NAG), an obligate cofactor for carbamyl phosphate synthetase I (CPSI) in the urea cycle. NAGS deficiency results in elevated levels of plasma ammonia which is neurotoxic. We report herein the first crystal structure of human NAGS, that of the catalytic *N*-acetyltransferase (hNAT) domain with *N*-acetyl-L-glutamate bound at 2.1 Å resolution. Functional studies indicate that the hNAT domain retains catalytic activity in the absence of the amino acid kinase (AAK) domain. Instead, the major functions of the AAK domain appear to be providing a binding site for the allosteric activator, L-arginine, and an *N*-terminal proline-rich motif that is likely to function in signal transduction to CPS1. Crystalline hNAT forms a dimer similar to the NAT-NAT dimers that form in crystals of bifunctional *N*-acetylglutamate synthase/kinase (NAGS/K) from *Maricaulis maris* and also exists as a dimer in solution. The structure of the NAG binding site, in combination with mutagenesis studies, provide insights into the catalytic mechanism. We also show that native NAGS from human and mouse exists in tetrameric form, similar to those of bifunctional NAGS/K.

## Introduction

*N*-acetylglutamate synthase (NAGS, EC 2.3.1.1) catalyzes the conversion of AcCoA and glutamate to CoA and *N*-acetylglutamate (NAG). In microorganisms and plants, NAG is further converted to NAG phosphate by NAG kinase (NAGK, EC 2.7.2.8) to continue the L-arginine biosynthetic pathway [1], [2]. However, in mammals, NAG has an entirely different role as the essential cofactor for carbamyl phosphate synthetase I (CPSI) in the urea cycle [3]. Perhaps because NAG plays different roles in lower organisms and mammals, L-arginine has opposing regulatory effects on their NAGS enzymes. In bacteria, particularly those that use the linear pathway for L-arginine biosynthesis, NAGS is feedback inhibited by the end product, L-arginine. Conversely, in mammals, L-arginine enhances the NAGS activity [3].

Phylogenetic analysis of NAGS protein sequences classifies them into two distinct types: bacteria-like, classic NAGS and vertebrate-like NAGS [4]. Most bacterial and plant NAGS belong to the former, with high sequence similarity to *Escherichia coli* NAGS. The second type includes not only vertebrate NAGS, but also fungal NAGS and NAGK, and bacterial bifunctional NAGS/K. Nevertheless, in spite of structural similarities of the second type NAGS of various species, it is still inhibited by L-arginine in microorganisms that utilize it.

Previously, we determined the structure of NAGS from *N. gonorrhoeae* (ngNAGS*)* and showed that this type of NAGS has a hexameric quaternary structure and that each subunit has two distinct domains: an *N*-terminal amino acid kinase (AAK) domain and a *C*-terminal *N*-acetyltransferase (NAT) domain [5]. The AAK domain has a structure similar to those of various *N*-acetylglutamate kinases (NAGK), but it is devoid of NAGK activity. It also has an L-arginine binding site similar to those in L-arginine sensitive NAGK structures [6]. The NAT domain has a typical GCN5-related NAT fold and a site that catalyzes NAG synthesis which is located >25 Å away from the L-arginine binding site [7]. We have also previously determined the structures of bifunctional NAGS/K from *Maricaulis maris* (mmNAGS/K) and *Xanthomonas campestris* (xcNAGS/K) [8]. Surprisingly, bifunctional NAGS/K oligomerizes to form a novel tetramer. Although the subunits of NAGS/K have similar structures to ngNAGS subunits with two distinct domains, their domain-domain linkers and relative domain orientations are different from those of ngNAGS. Inhibition by L-arginine of NAGS/K was proposed to result from changes in the relative orientations of AAK and NAT domains that close the AcCoA binding site.

Even though extensive efforts have been made to determine the mammalian NAGS structure, it has proven challenging because the complete protein is unstable in solution. We succeeded in obtaining stable and functional human NAGS NAT domain (hNAT) (residues 377–534) with NAG bound at 2.1 Å resolution. This structure and related mutagenesis experiments allowed us to define the catalytic mechanism. We have also confirmed by cross-linking and gel-filtration experiments that both human and mouse NAGS have tetrameric oligomeric structures similar to bifunctional NAGS/K. Therefore, the mechanisms that L-arginine uses to activate mammalian NAGS and inhibit bifunctional NAGS/K may be similar despite its disparate effects on the catalytic function.

## Results and Discussion

### Enzymatic Activity of the NAT Domain

**Figure 1:**
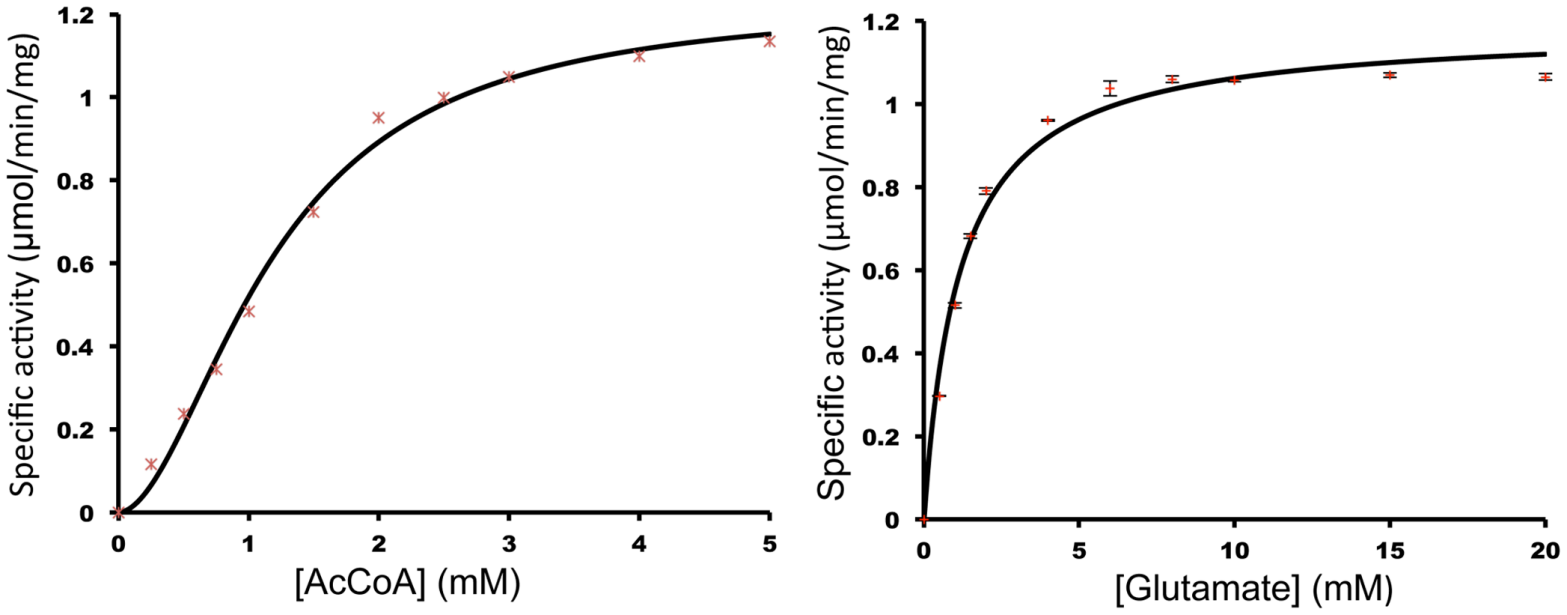
Biochemical properties of hNAT. A and B, dependence of enzyme activity on the concentration of AcCoA or L-glutamate. AcCoA or L-glutamate was varied in the range of 0.25–5.0 and 0.50–20.0, respectively, with L-glutamate and AcCoA fixed at 10 or 2.5 mM, respectively.

hNAT has detectable NAGS activity with a *V*_max_ value of 1.19±0.08 µmol/min/mg, but this value is approximately 6.6 fold lower than the specific activity of the full-length wild type hNAGS in the absence of L-arginine and 12.6 fold lower than the same in the presence of L-arginine (1 mM) under similar buffer conditions [9]. AcCoA and L-glutamate titration experiments (Figure 1) indicate that the absence of the AAK domain affects AcCoA binding affinity so that hNAT has a slightly higher apparent *K*_m_ value of 1.23±0.05 mM than the complete protein (0.94±0.04 mM). Glutamate binding appears to be stronger, with a *K*_m_ value of 1.18±0.03 mM lower than that of the complete protein (2.50±0.15 mM) in the absence of L-arginine, but close to that of 1.49±0.04 mM in the presence of L-arginine. AcCoA binding for hNAT shows significantly cooperativity with a Hill coefficient of 1.9±0.2, in contrast to the complete hNAGS which shows no cooperativity [9].

### Oligomerization State in Solution

**Figure 2:**
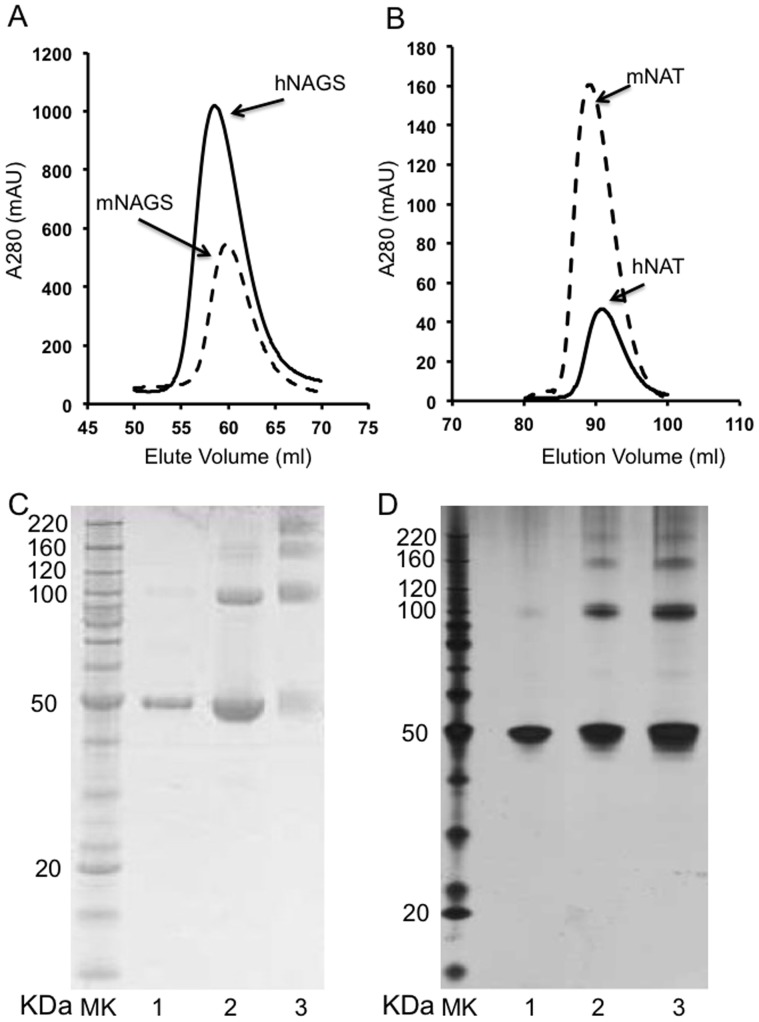
Oligomeric structure of mNAGS and hNAGS in solution. A: Analytic gel chromatography of mNAGS and hNAGS. Elution profiles of mNAGS and hNAGS are shown in dashed and solid lines, respectively. B: Analytic gel chromatography of mNAT and hNAT. Elution profiles of mNAT and hNAT are shown in dashed and solid lines, respectively. C: Cross-linking of mNAGS. Lanes 1; protein size markers; 2, mNAGS (2.5 µg) without cross-linking reagent; 3, mNAGS (2.5 µg) with cross-linking reagent, suberic acid bis(3-sulfo-N-hydroxysuccinimide ester) sodium salt; 4, mNAGS with cross-linking reagent, dimethyl suberimidate dihydrochloride. D: Cross-linking of mNAGS. Lanes 1; protein size markers; 2, mNAGS without cross-linking reagent; 3, mNAGS (1.5 µg) with cross-linking reagent, dimethyl suberimidate dihydrochloride; 4, mNAGS (4.5 µg) with cross-linking reagent, dimethyl suberimidate dihydrochloride.

To determine the states of oligomerization of both complete NAGS and the NAT domain in solution, cross-linking and analytic gel filtration experiments were performed. Cross-linking experiments using dimethyl suberimidate or suberic acid bis(3-sulfo-*N*-hydroxysuccinimide ester) sodium salt showed at least four bands on SDS-PAGE gels for both human and mouse complete NAGS, with molecular weights corresponding to oligomers of 1, 2, 3 and 4 subunits (Figure 2). Gel filtration experiments also demonstrated that complete hNAGS and mNAGS exist primarily as tetramers in solution. The molecular weights of mNAGS and hNAGS calculated from the standard curve are 199.2 and 220.1 KDa, respectively, consistent with tetramer molecular weights of 195.8 and 202.4 KDa for mNAGS and hNAGS, respectively. Molecular weights of mNAT and hNAT calculated from the standard curve are 36.2 and 36.1 kDa, respectively, implying they exist as dimers in solution since molecular weights of mNAT and hNAT dimers calculated based on the expected amino acid sequenced are 36.1 kDa matching the observed weight. The results are consistent with those for bifunctional mmNAGS/K and xcNAGS/K and imply that the hNAGS and mNAGS have similar tetrameric architectures to mmNAGS/K and xcNAGS/K in solution.

### Structure of hNAT with NAG Bound

**Figure 3:**
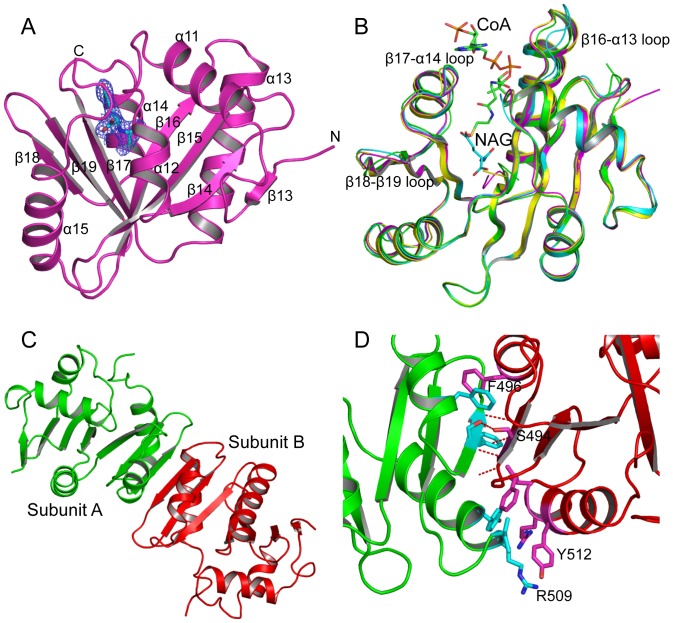
Structure of hNAT. A: Ribbon diagram of hNAT subunit structure. Bound NAG is shown as sky-blue sticks. The electron density map (2F_o_–F_c_) around bound NAG (contoured at 1.0 σ) is shown as blue cage. B: Superimposition of four hNAT subunits in asymmetric unit. The bound NAG is shown as sky-blue sticks. The proposed bound CoA is shown as green sticks. Subunits A, B, X and Y are shown in pink, yellow, green and blue ribbons, respectively. C: The hNAT molecular dimer. Subunits A and B are shown in green and red ribbons, respectively. D: Details of the interactions between subunits A and B. Side-chains of the residues in the interface are shown in sticks. Potential hydrogen bonding interactions are shown in red dashed lines.

The structure of hNAT (residue 377 to 534) was determined at 2.1 Å resolution and refined to *R*_work_ and *R*_free_ values of 18.4% and 24.4%, respectively (Table 1). The model has good geometry with 92.5% of the residues located inside the most favored area of a Ramachantran plot. Four copies of each subunit were identified in the asymmetric unit. The structures of the four subunits were not defined equally well with subunit A best defined, followed by subunit X, subunit B and subunit Y, with average temperature *B* factors of 35.0 Å^2^, 44.9 Å^2^, 54.2 Å^2^ and 78.1 Å^2^, respectively. Superimpositions of the four subunits result in RMS deviations of 0.4–0.8 Å (Table 2) with subunits A and B most similar, and subunit A and X most different. As shown in Figure 3B, the core secondary structures are very similar for all subunits, with the major differences in loop regions and terminal residues, which are usually highly flexible and easily affected by the different packing environments in the crystal. Since the structure of subunit A has the best quality, the structure description and discussion will be mainly based on this subunit.

**Table 1:** Data collection and refinement statistics.

Data collection	
Bound ligands	NAG
Space group	*P*4_3_2_1_2
Wavelength (Å)	1.0
Resolution (Å)	50–2.10 (2.14–2.10)^a^
Unit-cell parameters (Å)	*a = b = *116.1
	*c = *109.7
Measurements	600,087
Unique reflections	83,202 (4,107)
Redundancy	7.2 (5.1)
Completeness (%)	99.9 (99.1)
<*I/σ*(*I*)>	21.7 (1.7)
* R*_merg_(%)^b^	8.1 (65.6)
**Refinement**	
Resolution range (Å)	40–2.10 (2.15–2.10)
No. of protein atoms	4,963
No. of water atoms	266
No. of hetero atoms	52
Rmsd of bond lengths (Å)	0.008
Rmsd of bond angle (°)	1.1
* R*_work_ (%)^c^	18.5 (25.2)
* R*_free_ (%)^d^	24.4 (31.5)
Ramachandran plot (%)	
Favored	92.5
Allowed	7.3
Generous	0.2
Disallowed	0.0

a Figures in brackets apply to the highest-resolution shell.

b *R*_merg_ = Σ*_h_*Σ*_i_*|*I*(*h*,*i*)*-<I*(*h*)*>*|/Σ*_h_*Σ*_i_I*(*h*,*i*), where *I*(*h*,*i*) is the intensity of the *i*th observation of reflection *h*, and<*I*(*h*)> is the average intensity of redundant measurements of reflection *h*.

c *R*_work_ = Σ*_h_*||*F*_obs_| *–* |*F*_calc_||/Σ*_h_* |*F*_obs_|.

d *R*_free_ = Σ*_h_*||*F*_obs_| *–* |*F*_calc_||/Σ*_h_* |*F*_obs_| for 5% of the reserved reflections.

**Table 2:** RMSD values (Å) among different subunit within NAG bound structure and with the bifunctional mmNAGS/K native structure.

Subunit	A	B	X	Y
A	**1.08** a	0.42	0.76	0.60
B	**1.19**		0.69	0.61
X	**1.05**			0.48
Y	**1.04**			

a The values in bold are RMSD of the subunit A in hNAT structure with subunits in mmNAGS/K NAT domain structure (PDB 3S6H).

Each subunit has a central seven-strand β-sheet arranged as a *V*-shaped structure with three anti-parallel β-strands in the *C*-terminal arm and four anti-parallel β-strands in the *N*-terminal arm (Figure 3A). The central β-sheet is flanked by five helices with three helices on one side and four helices on the other. The structure has a typical fold of GCN5-related *N*-acetyltransferase and is similar to the NAT domain structure of the bifunctional NAGS/K from *M. maris* (Table 2).

### Dimerization

Even though four subunits were identified in an asymmetric unit, the PISA server [10] indicated that the stable molecule is dimer. Subunit A and subunit B form a molecular dimer. The molecular dimers for subunit X and subunit Y were generated via crystallographic two-fold symmetries, respectively. At each dimer interface (A-B, X-X or Y-Y), the *C*-terminal arm from one subunit interacts with the C-terminal arm from the other subunit to form a continuous 6-strand antiparallel β-sheet, similar to the NAT-NAT domain interaction observed in the mmNAGS/K structure (Figure 3C). This extensive interface has a buried interface of 1477 Å^2^. The interactions in this interface involve extensive main-chain (Asp490, Ser492 and Ser494) and side-chain (Ser494) hydrogen bonding interactions, π-π interactions (Phe496–Phe496′), π-cation interactions (Tyr512–Arg509′) and other hydrophobic interactions (Figure 3D).

### NAG Binding Site

**Figure 4:**
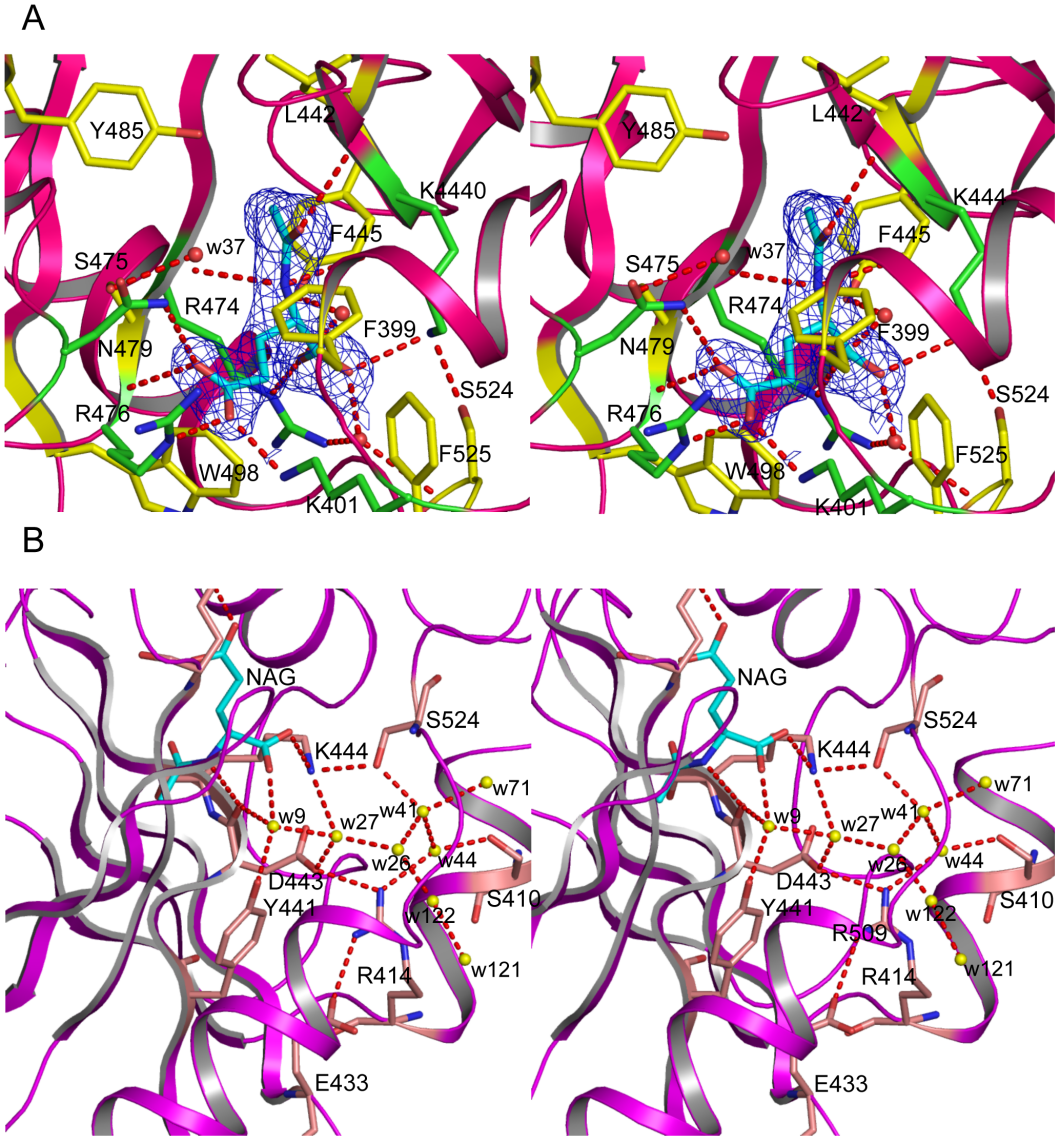
NAG binding site. A: Stereo diagram of NAG binding site. The bound NAG is shown in sky-blue sticks. The side-chains involved in hydrogen bonding interactions with NAG are shown in green sticks. The side-chains of other surrounding residues are shown in yellow sticks. The water molecule (w37) is shown in red ball. The electron density map (2F_o_–F_c_) around bound NAG (contoured at 1.0 σ) is shown as blue cage. Potential hydrogen bonding interactions are shown in red dashed lines. B: Stereo diagram of “water wire” channel. The bound NAG is shown in sky-blue sticks. Water molecules are shown in yellow balls. Residues involved in hydrogen bonding interactions are shown in brown sticks. Potential hydrogen bonding interactions are shown in red dashed lines.

The electron density map was readily interpretable with NAG visible at the enzyme active site. NAG binds in a cavity surrounded by the central β-sheet (strands β16 and β17), the loop connecting helices α11 and α12, and the *C*-terminal segment (Figure 3A). The side-chains of five residues, Lys444 from the strand β16, Arg474 and Arg476 from strand β17, Asn479 from the loop connecting β17 and α14 and Lys401 from the loop connecting helices α11 and α12 are involved in hydrogen bonding to NAG (Figure 4A, Table 3). The main-chain O of Asp443 and Arg473 and the main-chain N of Phe445 and Arg476 are also involved in positioning NAG by anchoring different functional groups of NAG. The side-chains of Phe399, Leu442, Trp498 and Phe525 form hydrophobic interactions with the side-chain of NAG holding the side-chain in place. These extensive hydrogen bonding and hydrophobic interactions place NAG or L-glutamate in the right position and orientation to facilitate the catalytic reaction and define the specificity of hNAGS. All these residues are either invariant (Phe399, Leu442, Asp443, Lys444, Phe445, Arg474, Arg476, Asn479, Trp498 and) or conservatively substituted (Lys401 and Phe525) in vertebrate-like NAGS. However, in contrast to bacterial-like NAGS such as *Neisseria gonorrhoeae* NAGS [5], hNAGS uses different residues to bind NAG, supporting the hypothesis that the NAT domains of vertebrate-like and bacterial-like NAGS evolved from different ancestors.

**Table 3:** Interactions between *N*-acetyl-L-glutamate and protein atoms.

Arginine	Protein	Distance (Å)			
		Subunit A	Subunit B	Subunit X	Subunit Y
N2	Asp443 O	3.37	3.41	3.29	3.29
	Arg474 O	3.23	3.19	3.23	3.33
O7	Phe445 N	2.96	3.00	3.04	3.24
OXT	Lys444 NZ	3.08	2.61	2.97	3.46
	Wat258^a^ O	2.47	3.37		
O	Arg474 NE	2.94	3.16	2.96	2.87
	Wat258 O	3.22	2.47	2.47	
	Wat9 O	2.64			
OE1	Asn479 ND	2.96	3.48	4.95^b^	3.43
	Arg476 N	2.98	3.10	4.22^b^	3.19
OE2	Lys401 NZ	2.64	3.31	2.28	4.01^b^
	Arg476 NE	2.61	3.69	2.77	3.53^b^

a Water numbering for subunit A only.

b The distances are too far away for hydrogen bonding interactions.

### CoA Binding Site

**Figure 5:**
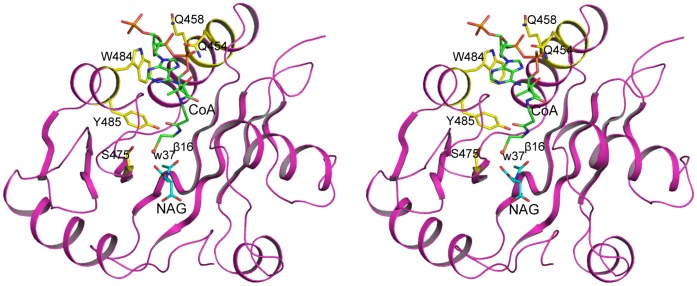
Stereo diagram of the proposed CoA binding site. The proposed bound CoA is shown in green sticks. The bound NAG is shown in sky-blue sticks. Side-chains of residues that potentially hydrogen bond to CoA are shown in yellow sticks. The water molecule (w37) that occupies the similar position of thiol S of CoA is shown in a red ball.

Even though 10 mM CoA was present in the crystallization solution, no continuous electron density corresponding to CoA was observed in the “*V*-shaped” groove where the pantetheine moiety of CoA usually binds, probably due to suboptimal conditions for CoA binding. However, the unambiguous identification of NAG in the expected site suggests that CoA is likely to bind in a site similar to those found in other GCN5-related acetyltransferases. Structural comparison of hNAT with the NAT domain of ngNAGS, which has both substrates bound, allows identification of the CoA binding site and development of a model of the catalytic mechanism. Superimposition of the hNAT with the NAT domain of ngNAGS clearly indicates that the pantetheine moiety of CoA interacts with the protein through hydrogen bonds with the main-chain nitrogen of Phe445 and the main-chain oxygen of Val447 from β16, in a way similar to an anti-parallel β-sheet (Figure 5). The thiol sulfur is oriented in a position within hydrogen bonding range of the side-chains of Tyr495 and Ser475. One water molecule (w37) was identified occupying the thiol sulfur position in the present structure, 3.4 Å away from the acetyl carbon and perpendicular to the acetyl group plane of NAG. The structure is consistent with hNAGS using a one-step direct attack catalytic mechanism to transfer the acetyl group from AcCoA to the amino group of L-glutamate, as is the case for most members of the GCN5-related NAT family. The pyrophosphate moiety appears to be in proximity to the sequence Gln452–Gly453–Gln454–Gly455–Ser456–Gly457–Gln458, which conforms to (Arg/Gln)-Xaa-Xaa-Gly-Xaa-(Gly/Ala) motif for AcCoA recognition and binding in known GCN5-related *N*-acetyltransferases [11]. Because of the absence of AcCoA or CoA binding, this part of structure varied significantly among different subunits (Figure 3B). Upon AcCoA or CoA binding, the structure needs to adjust in order to bind the pyrophosphate moiety. The side-chains of Gln454 and Gln458 of this motif, as well as Trp484, seem to be involved in positioning CoA. The adenosine moiety of CoA is located on the surface of the protein, as seen in other GCN5-related NAT structures [12].

### Comparison of the NAT Domain Structures of Human NAGS and mmNAGS/K

**Figure 6:**
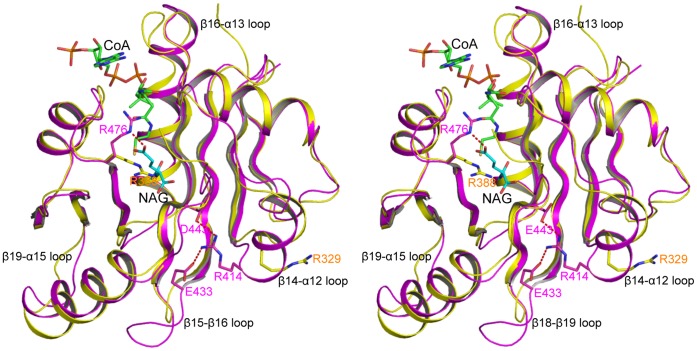
Superimposition of hNAT with the NAT domain of subunit X of mmNAGS/K. The structure of hNAT is shown in pink ribbons. The structure of the NAT domain of subunit X of mmNAGS/K is shown in yellow ribbons. The bound NAG is shown in sky-blue sticks. The proposed bound CoA is shown in green sticks. Residues that are mentioned in text are shown in sticks.

The overall hNAT structure is similar to that of mmNAGS/K (Figure 6, Table 2) and can be aligned with an RMS deviation of ∼1.0 Å, even though different subunits in mmNAGS/K have different relative orientations of the AAK and NAT domains [8]. The major structural differences occur in the loop regions (α12–β14, β16–α13, β15–β16 and β19–α15 loops). The significant conformational changes in the pyrophosphate moiety binding motif in the loop connecting β16 and α13 demonstrate the high flexibility in this region in the absence of AcCoA binding, as shown in the variation among different subunits. The conformational changes of the side-chain position of Arg476 may be functionally significant. In all mmNAGS/K subunits, the side-chain of Arg388 (the equivalent residue of Arg476) points outwards (Figure 6) whereas in the NAG bound hNAT structure, this side-chain moves towards the substrate binding site to anchor the γ-carboxyl group of NAG. Another interesting difference is in the α12–β14 loop in which two more residues are present in hNAT compared to mmNAGS/K. The side-chain of Arg414 of this loop swings towards the NAG binding site to form a hydrogen bond with the side-chains of Asp433 and Asp443. At least 8 nearby water molecules link the amino nitrogen of NAG to the side-chains of Tyr441, Asp443, Lys444, Ser524, Arg414 and Ser410 in a string that extends to the protein surface (Figure 4B). As proposed for serotonin *N*-acetyltransferase [13], this chain of water molecules may be a “proton wire” to ferry away a proton from the substrate and facilitate a nucleophilic attack on AcCoA.

### Implications for Catalysis

To confirm the catalytic mechanism, several residues in this site were selected for biochemical studies. Tyr485, the equivalent residue of Tyr397 in mmNAGS/K and Tyr405 in xcNAGS/K, appears to act as a catalytic acid that donates a proton to the thiol group of CoA, playing an important role in the catalytic reaction (Figure 4A). This equivalent tyrosine could be identified in most GCN5-related acetyltransferases [14]. Indeed, the Y485F mutant showed 10 fold lower catalytic activity than wild-type protein (Table 4).

**Table 4:** Enzyme activities of hNAT and active site mutants.

Sample	Activity (µmoles/min/mg)^a^
WT	1.05±0.01
WT+L-arginine (1 mM)	1.04±0.01
Y485F	0.078±0.003
Y441F	0.857±0.004
N479A	0.154±0.002

a Means ± standard errors of means (*n* = 3) are shown.

Since the α-amino group of L-glutamate has a pKa value that is close to 10, it seems clear that amine deprotonation must precede the acetyl group transfer. The highly conserved Tyr441 located in the water channel that connects to the α-amino group (see previous section), is positioned to play a role as the catalytic base in proton removal. The lower activity of Y441F mutant is consistent with this catalytic role of this tyrosine. The 7 fold lower activity for N479A mutant confirmed that it is a key residue to bind L-glutamate as found in the present structure (Figure 4A).

### Mechanism of L-arginine Regulation

Since hNAGS and mNAGS have similar oligomeric structures (tetramers), as demonstrated in our cross-linking and gel-filtration experiments (Figure 2), and the dimer architecture of hNAT is similar to the NAT-NAT domain interface in mmNAGS/K (Figure 3C), the quaternary structure of hNAGS and mNAGS is likely to be similar to that of bifunctional mmNAGS/K. L-arginine binding may also cause rotation of the NAT domain towards to the AAK domain in mammalian NAGS, but to a lesser degree than in mmNAGS/K to allow AcCoA to bind to the active site, because the domain linkers of mammalian NAGS and bacterial bifunctional NAGS/K consist of different amino acids. The enhancement of NAGS activity by arginine in mammalian NAGS may be caused by increasing the AcCoA binding affinity via favorable hydrogen bonding interactions of residues in the AAK domain, facilitated by the conformational changes induced upon arginine binding.

### Roles for the AAK Domain

The major role of NAGS in the urea cycle is to produce the essential cofactor, NAG, to activate CPSI. Among the three mitochondrial enzymes of the urea cycle, NAGS is the least abundant by far, thousands fold lower than CPSI and OTCase. Since the NAT domain alone has catalytic activity and is stable, an interesting question arises: why has the AAK domain remained intact through evolution? Even though activity assays demonstrate that the AAK domain enhances NAGS activity 6 to 12 fold, this may not be the major reason since an increase in enzyme abundance could compensate for lower activity. A more probable explanation is a regulatory role of the AAK domain in urea cycle flux. Complete hNAGS has two extra features relative to hNAT that may play a role in regulating urea cycle flux. First, the binding of L-arginine enhances NAGS activity and the arginine-binding site that is located in the AAK domain is conserved in NAGS across phyla [4]. In microorganisms, arginine biosynthesis is regulated via this arginine binding site because bound L-arginine is an allosteric inhibitor of NAGS activity [7]. It is therefore reasonable to assume that in mammals, urea cycle flux can be rapidly enhanced via increased NAGS activity by L-arginine binding at this site. Our *N*-carbamylglutamate (NCG) clinical trial experiments demonstrated that NCG could enhance urea cycle flux even in healthy individuals [15], implying that under normal conditions, CPSI is not fully saturated with NAG. Increasing NAG production will therefore increase urea production by activating additional CPSI molecules. Second, the presence of a proline-rich region in the *N*-terminal sequence of mammalian NAGS (AAK domain) may be important in interacting with CPSI to facilitate NAG translocation from NAGS to CPSI. Proline-rich motifs often serve as targets for protein recognition and interaction since they are recognized by many proteins, including important signaling proteins such as Src homology 3 [16], the WW domain of a kinase-associated protein [17], Enabled/VASP (EVH1) [18] and ubiquitin-E2-like variant (UEV) domain of the tumor maintenance protein Tsg101 [19]. Crystal structures of these motifs demonstrate that they are usually exposed to solvent and have a collagen-like polyproline type II (PPII) extended conformations. Most of these PPII motifs are involved in protein-protein interactions that seem important for signal transduction and metabolic regulation [20].

### Clinical Implications

This study demonstrates that hNAT is stable and has catalytic activity. The results are consistent with previous observations about potential effects of hNAGS missense mutations in patients. Missense mutations in the AAK domain are usually “milder” than mutations in the NAT domain and are usually associated with “late-onset” clinical presentation [21]. All missense mutations associated with neonatal-onset, severe manifestations identified so far are located in the NAT domain. While the NAT domain plays a key role in NAGS activity and is mainly encoded by the last three exons of the human gene, the mitochondrial peptide signal and the proline-rich variable segment are encoded by the first exon of the gene [22]. Thus, a putative nonsense and out of frame mutations in the AAK domain (exons 2–4) might be rescued by exon skipping therapy that could restore the correct reading frame for encoding the NAT domain.

## Materials and Methods

### Cloning and Protein Expression and Purification

Human NAGS (hNAGS), mouse NAGS (mNAGS), hNAT and all mutants were expressed and purified as described previously [5]. Briefly, the proteins were expressed in *E. coli* BL21(DE3) cells (Invitrogen) and purified with nickel affinity and Histrap SP columns (GE Healthcare). Protein purity was verified by SDS/PAGE gel and protein concentration was measured with a Nano-drop 1000 spectrophotometer (Thermo Scientific). The extinction coefficient obtained from the ExPASy web server (http://web.expasy.org/protparam/) was used to calculate protein concentrations. The protein was stored at 253 K in a buffer containing 50 mM Tris-HCl, pH 7.4, 50 mM NaCl, 10% glycerol, 5 mM β-mercaptoethanol, and 1 mM EDTA.

### Site-directed Mutagenesis

Site-directed mutant DNA sequences encoding hNAT were created using primers containing the desired mutations and the QuikChange Mutagenesis Kit according to the manufacturer’s protocol (Strategene). The sequences of mutant DNA sequences were verified by DNA sequencing.

### Activity Assay

Enzymatic activity was assayed using the method described previously [23]. A stable isotope dilution method using liquid chromatography mass spectrometry (LC–MS) to measure NAG production was adapted. Each assay was performed in a 100 µl solution containing 50 mM Tris, pH 8.5, 10 mM glutamate and 2.5 mM AcCoA. The reaction was initiated by the addition of purified recombinant enzyme (20 µg), and the mixture was incubated at 303 K for 5 min and quenched with 100 µl of 30% trichloroacetic acid containing 50 µg of *N*-acetyl-[^13^C_5_]glutamate (^13^C-NAG) as an internal standard. Precipitated protein was removed by micro-centrifugation. The supernatant (10 µl) was submitted to LC-MS (Agilent) analysis. The mobile phase consisted of 92% solvent A (1 ml trifluoroacetic acid in 1 L water) and 8% solvent B (1 ml trifluoroacetic acid in 1 L of 1∶9 water/acetonitrile) and the flow rate was 0.6 ml/min. Glutamate, NAG, and ^13^C-NAG were detected and quantified by selected ion monitoring mass spectrometry.

AcCoA and glutamate titration experiments were carried out with AcCoA or L-glutamate concentration varied in the range of 0.25–5.0 and 0.5–20 mM, respectively, and L-glutamate or AcCoA concentration fixed at 10 and 2.5 mM, respectively. The L-glutamate titration data were fit to Michaelis-Menten kinetics, while AcCoA titration data were fit to sigmoidal kinetics (*V* = *V*_max_ [AcCoA]*^n^*/([AcCoA]*^n^*+*K*_m_ *^n^*), where *V*_max_ is maximum activity, *K*_m_ is half-maximum activity and *n* is the Hill coefficient, using the program GNUPLOT.

### Cross-linking Experiment

Cross-linking experiments were performed using the protocol described by Davies and Stark [24]. mNAGS (2.5 µg ) and hNAGS (1.5 and 4.5 µg) were incubated with the cross-linking reagent dimethyl suberimidate (4.5 µg) or suberic acid bis(3-sulfo-*N*-hydroxysuccinimide ester) sodium salt (9.0 µg) in 10 µl solution containing 200 mM triethanolamine, pH 8.25 for three hours at 298 K. Samples of mNAGS with and without cross-linking reagent were subjected to sodium dodecyl sulfate polyacrylamide get electrophoresis (NuPAGE 4–12% Bis-Tris gel) in MES SDS buffer (50 mM MES, 50 mM Tris base, 0.1% SDS, 1 mM EDTA, pH 7.3) and stained with Coomassie blue. Samples of hNAGS with and without cross-linking reagent were subjected to sodium dodecyl sulfate polyacrylamide get electrophoresis (NuPAGE 4–12% Bis-Tris gel) in MES SDS buffer (50 mM MES, 50 mM Tris base, 0.1% SDS, 1 mM EDTA, pH 7.3) and stained with silver. Size marker controls consisted of proteins with defined molecular weights of protein standards purchased from Invitrogen.

### Gel-filtration Chromatography

Molecular weight of mNAGS and hNAGS were determined with a Superdex 200 HR 10/30 column (Amersham Biosciences) as previously described [5]. The running buffer contains 100 mM NaH_2_PO_4_ pH 7.4, 150 mM NaCl, 10% glycerol, 1 mM β-mercaptoethanol. Thyroglobulin (669 kDa), ferritin (440 kDa), ngNAGS (296.7 kDa), mmNAGS (200.5 kDa) and aldolase (158 kDa) were used as protein standards.

Molecular weights of hNAT and the NAT domain of mouse NAGS (mNAT) were determined similarly, but with different protein standards of ovalbumin (43 kDa), albumin (67 kDa), chymotrypsinogen A (25 kDa) and ribonucleause (13.7 kDa).

### Crystallization

Crystals were grown by the sitting-drop, vapor-diffusion method. Before crystallization, the purified protein (∼20 mg/ml) was treated with thrombin (50 units) overnight at 277 K to remove the his-tag, then incubated with 10 mM CoA, and 20 mM NAG for 30 min. Screening of crystallization conditions was performed using sitting-drop vapor diffusion in 96-well plates (Hampton Research) at 291 K by mixing 2 µl of the protein solution with 2 µl of the reagent solution from the sparse matrix Crystal Screens 1 and 2, and Index Screen (Hampton Research). The best crystals were grown from a reservoir solution containing 100 mM Bis-tris, pH 6.5, 35% PEG3350. Crystals were stick-shaped and took 2–3 days to reach a maximal length of 0.6 mm.

### Data Collection and Structure Determination

Crystals were transferred from the crystallization plate to a well solution supplemented with 25% glycerol and then frozen directly by liquid nitrogen. Diffraction data were collected at beamline 22-ID equipped with MAR300 CCD at the Advanced Photon source (APS), Argonne National Laboratory, USA. All data were processed using the HKL2000 package [25]; statistics are summarized in Table 1. The structure was solved by molecular replacement using Phaser [26], [27] based on the NAT domain of mmNAGS/K structure of subunit X as a search model. After several cycles of refinements with Phenix [28] and model adjustments with Coot [29], NAG was visible in the electron density map and was built into the model. In the last run of the refinement, the translation/liberation/screw parameters were included and refined [30]. Two groups per subunit were selected according to the *N*-terminal arm (residues 375–469) and the *C*-terminal arm (470–527). Final *R* and *R*_free_ values were 18.4% and 24.4%, respectively. Refinement statistics for the final refined model are given in Table 1. The final refined coordinates for NAG bound hNAT and its structure factors have been deposited in RCSB Protein Data Bank with accession code 4K30 and provided as Supplemental Materials.

## Supporting Information

File S1 Coordinate file for the described structure.
(PDB)

File S2 Structure factors for the described structure.
(CIF)
